# Transcatheter drug delivery through bronchial artery for COVID-19: is it fiction or could it come true?

**DOI:** 10.1186/s41747-020-00171-4

**Published:** 2020-07-06

**Authors:** Mohamed M. A. Zaitoun, Mohammad Abd Alkhalik Basha, Ali Hassan Elmokadem

**Affiliations:** 1 Diagnostic Radiology Department, Faculty of Medicine, Zagazig University, Zagazig, Sharqiyah Egypt; 2 Diagnostic Radiology Department, Faculty of Medicine, Mansoura University, Mansoura, Egypt

**Keywords:** Angiography, COVID-19, Bronchial arteries, Pandemics, Severe acute respiratory syndrome coronavirus 2 (SARS-CoV-2)

## Abstract

More than 1,200 active or recruiting clinical trials for novel coronavirus disease 2019 (COVID-19) treatments and vaccines are registered. Many drugs have shown promise for treatment of COVID-19. Nevertheless, up to date, no drugs have been confirmed as a definitive treatment for COVID-19. Trials such as the SOLIDARITY and RECOVERY are ongoing, and first results were announced in favour of therapy with dexamethasone with a significant trend showing greatest benefit among those patients requiring ventilation. The drawbacks of these trials include exposing the patients to drugs with well-documented systemic adverse effects or unknown complications of novel therapies without proof of clinical benefit. We present here the hypothesis that bronchial artery infusion could be an alternative for systemic drug infusion in COVID-19 trials with superadded benefits of high drug concentration and low systemic adverse effects. The concept of this idea has many uncertainties and no current clinical data to support. Perhaps, the technique should be first applied in animal models to determine its safety and calculate the effective dose of the drugs. Guidelines and reviews of pharmacotherapy for COVID-19 should be implemented for this fiction to come true.

## Key points

- COVID-19 is a global pandemic.
- Many active clinical trials of COVID-19 treatments and vaccines are registered.
- Bronchial artery infusion (BAI) could be an alternative for systemic drug infusion in COVID-19 trials.

The novel coronavirus disease 2019 (COVID-19) is a highly infectious disease caused by a severe acute respiratory syndrome coronavirus (SARS-CoV-2) which started in Wuhan, China, in December 2019. The World Health Organization (WHO) declared COVID-19 a global pandemic on March 11, 2020 [1]. Overall, there have been 8,242,999 confirmed cases and 445,535 deaths distributed across the globe as of June 18, 2020 [2]. While COVID-19 pandemic continues to spread, causing exponential morbidity and mortality, and threatening economies, the world is racing to find safe and effective treatments.

More than 1,200 active or recruiting clinical trials for COVID-19 treatments and vaccines are registered [3]. Several agents are being investigated as potential therapies for COVID-19. Many drugs have shown high promise for treatment of COVID-19 such as immunomodulatory, anti-inflammatory, antiviral, and antiparasitic agents. Nevertheless, up to date, no drugs have been confirmed as a definitive treatment for COVID-19.

On March 20, 2020, the WHO and partners announced the SOLIDARITY trial [4]. The SOLIDARITY trial is comparing three treatment options against the standard of care, to assess their relative effectiveness against COVID-19 and discover whether any of these drugs slow disease progression or improve survival. Treatment options under the study are the following: (i) remdesivir, a previously tested Ebola treatment; (ii) lopinavir/ritonavir, a licensed treatment for the human immunodeficiency virus (HIV) infection; and (iii) interferon-β-1a, used to treat multiple sclerosis. The chloroquine and hydroxychloroquine arm was removed from the SOLIDARITY trial on June 17, 2020 [4].

The “Randomized evaluation of COVID-19 therapy” (RECOVERY) trial [5] is another randomised controlled clinical trial including 11,500 patients who have been randomised to the following treatment arms: (i) lopinavir-ritonavir; (ii) low-dose dexamethasone, an anti-inflammatory steroid; (iii) azithromycin, an antibiotic; (iv) tocilizumab (an anti-inflammatory treatment); (v) convalescent plasma (collected from donors who have recovered from COVID-19); and (vi) hydroxychloroquine (which has been stopped). Overall, the 28-day mortality rate was reduced by 17% using dexamethasone with a highly significant trend showing greatest benefit among those patients requiring ventilation (test for trend, *p <* 0.001) [5]. The drawbacks of these trials include exposing the patients to drugs with well-documented systemic adverse effects or unknown complications of novel therapies without proof of clinical benefit.

Based on the first-pass effect of transcatheter therapy, we can hypothesise that *bronchial artery infusion* could be an alternative for systemic drug infusion in COVID-19 trials with superadded benefits of high drug concentration and low systemic adverse effects. Studies demonstrated that the bronchial artery is the main feeding artery for lung tumours. Hence, bronchial artery infusion is used as a palliative treatment option for primary and metastatic lung cancers [6]. Additionally, transcatheter therapy for purposes other than tumour management was reported. Transcatheter arterial steroid injection therapy prevented the progression of severe acute hepatic failure to a fatal stage of fulminant liver failure in 13 out of 17 patients [7].

However, the situation is different for COVID-19 as the exact pathophysiologic mechanism of ground-glass opacities is still unclear, and the possibility of being fed by bronchial arteries is not proven as in case of lung neoplasms. Furthermore, the blood flow into the lung in cases of pneumonia might be reduced as reported before in experimental animals [8]. The other major obstacle is how to prepare the angiographic suite for COVID-19 to control the spread of infection to the intervention radiology staff and other patients. Ierardi et al. [9] reported strict preprocedural, intraprocedural, and postprocedural workflow measures in the setting of an IR unit that is highly exposed to COVID-19. These safe practices may raise the equipment cost, prolong procedural times, and increase technical difficulties.

The search for successful therapies for COVID-19 infection is a challenging process. To the best of our knowledge, no previous reports or registered trial was proposing the transcatheter drug delivery through the bronchial artery to treat COVID-19. Proposed drugs for transcatheter therapy are drugs evaluated by SOLIDARITY and RECOVERY trials in addition to anticoagulant therapy that were associated with a better outcome.

The concept of this idea has many uncertainties and no current clinical data to support. Perhaps, the technique should be first applied in animal models to determine its safety and calculate the effective dose of the drugs. Guidelines and reviews of pharmacotherapy for COVID-19 should be implemented for this fiction to come true.

## Data Availability

Not applicable

## Abbreviations

COVID-19: Coronavirus disease 2019
SARS-CoV-2: Severe acute respiratory syndrome coronavirus
